# Early and sex-specific dynamic changes of the modified Glasgow Prognostic Score correlate with survival and immunotoxicities in patients undergoing allogeneic hematopoietic stem cell transplantation

**DOI:** 10.3389/fimmu.2026.1894926

**Published:** 2026-08-17

**Authors:** Hannah Thurisch, Kathrin C. Althoff, Katharina Leuchte, Wajma Shahbaz, Maximilian A. Funk, Martin E. Kirmaier, Amar Hadzic, Sabine Oganesian, Adriano Carboniero, Ludovica Vona, Caroline Geier, Lena Muschik, David M. Cordas dos Santos, Michael von Bergwelt-Baildon, Udo Holtick, Christof Scheid, Sebastian Theurich

**Affiliations:** 1 Cancer and Immunometabolism Research Group, Gene Center, Ludwig-Maximilians-Universität (LMU) München, Munich, Germany; 2 Department of Medicine III and Comprehensive Cancer Center Munich, LMU University Hospital, Munich, Germany; 3 German Cancer Consortium (DKTK), Munich Site, and German Cancer Research Center, Heidelberg, Germany; 4 Bavarian Cancer Research Center (BZKF), LMU University Hospital, Munich, Germany; 5 Department I of Internal Medicine, Medical Faculty and University Hospital of Cologne, University of Cologne, Cologne, Germany; 6 Department of Otorhinolaryngology, University of Lübeck, Lübeck, Germany; 7 National Center for Cancer Immune Therapy (CCIT-DK), Department of Oncology, Copenhagen University Hospital Herlev, Herlev, Denmark

**Keywords:** allogeneic hematopoietic stem cell transplantation, graft-versus-host disease, immunonutritional status, inflammation, metabolism, risk assessment

## Abstract

Risk assessment before allogeneic hematopoietic stem cell transplantation (alloHSCT) is essential to estimate treatment-related morbidity and mortality; the hematopoietic cell transplantation-comorbidity index (HCT-CI) is widely used. The modified Glasgow Prognostic Score (mGPS), reflecting systemic inflammation and nutritional status, is a validated prognostic marker in solid tumors, but ill-defined in alloHSCT. We evaluated the prognostic impact of mGPS before and early after alloHSCT on overall survival (OS), non-relapse mortality (NRM), and acute graft-versus-host disease (aGvHD). We analyzed 442 consecutive alloHSCT patients (2012–2017, University Hospital Cologne). Outcomes were assessed by mGPS pre-transplant (preTx), at day+30, and by dynamic changes (Δ^30^mGPS). Subgroup analyses included transplant characteristics and sex. Patients with mGPS 2 preTx had inferior OS (HR 2.32, p<0.0001) and NRM (HR 2.31, p=0.002). Moreover, Δ^30^mGPS changes indicated poorer OS and NRM rates more strongly (Δ^30^mGPS_1→2vs0→1_: OS: HR 2.85, p=0.02; NRM: HR 3.99, p=0.05). Findings regarding aGvHD were conflicting. Multivariable analysis confirmed mGPS as an independent prognostic factor for survival. Sex-specific analyses revealed distinct risk patterns. These findings highlight the prognostic relevance of the immunonutritional status before and early after alloHSCT, warranting prospective studies to further validate the mGPS for refined risk stratification.

## 1 Introduction

Allogeneic hematopoietic stem cell transplantation (alloHSCT) is a well-established and potentially curative treatment for various hematologic malignancies and disorders (1). Its broader application, however, remains limited by transplant-related toxicities, particularly acute graft-versus-host disease (aGvHD) and infections, which are the leading causes of non-relapse mortality (NRM). Despite advances in conditioning regimens, aGvHD prophylaxis, and infection management, NRM rates remain at 20–32%, with aGvHD grades II–IV occurring in 30–50% of patients, underscoring the need for a better understanding of NRM risk factors and potential underlying mechanisms (2–5).

The hematopoietic cell transplantation-comorbidity index (HCT-CI) is widely used to estimate treatment-related morbidity and mortality following alloHSCT, but captures metabolic vulnerability only incompletely, considering obesity (body mass index (BMI) >35 kg/m²) and diabetes as the only factors in this category (6). In contrast, impaired nutritional status and systemic inflammation have emerged as important host-related risk factors across cancer entities, particularly studied in solid tumors (7, 8). A meta-analysis by Mantzorou et al. highlighted the paucity of research addressing malnutrition and chronic inflammation in hematologic malignancies beyond BMI (7). While BMI has been shown to be associated with alloHSCT outcomes (1, 9, 10), contradictory and multifaceted results suggest more complex underlying mechanisms, including increased catabolism and a pathologic immunonutritional status.

The modified Glasgow Prognostic Score (mGPS) was introduced as a survival predictor in solid malignancies and stratifies patients into three risk groups based on serum albumin and C-reactive protein (CRP) reflecting nutritional status and systemic inflammation, respectively (11, 12). In alloHSCT, evidence remains scarce and is limited to a single study focusing exclusively on pre-transplant mGPS (preTx mGPS), which nevertheless demonstrated prognostic relevance for overall survival (OS) and NRM (3, 8).

Although several prognostic or predictive scores have been developed in alloHSCT (13), they largely rely on static baseline variables and do not incorporate early post-transplant dynamics or sex-specific differences. Data on immunonutritional biomarkers in the post-transplant phase remain limited. Prior work has shown a strong correlation between low serum albumin levels, albumin deficiency at day+30 and increased NRM, underscoring the clinical relevance of dynamic nutritional parameters (2). Parallel to these developments, recent advances in machine learning (ML) and computational intelligence have substantially expanded personalized risk prediction and dynamic modeling in medicine, complementing traditional prognostic scores (14–16). Nevertheless, simple and clinically interpretable biomarkers such as the mGPS remain valuable in routine practice.

Despite increasing evidence highlighting the relevance of the immunonutritional status in alloHSCT, the prognostic value of the mGPS, particularly its early post-transplant dynamics and potential sex-related differences, remains insufficiently characterized. Addressing knowledge gaps may further establish the mGPS as a clinically feasible tool for risk stratification and individualized supportive care after alloHSCT. Therefore, we aimed to evaluate the prognostic value of the mGPS before transplantation and its dynamic changes within the first 30 days after alloHSCT in relation to survival and transplant-related toxicities, including aGvHD. Given known sex differences in body composition and inflammatory responses (17), we additionally explored whether associations of preTx mGPS, day+30 mGPS and early mGPS dynamics with OS, NRM and aGvHD differed between sexes. Our findings contribute to the current knowledge by providing the first evaluation of early mGPS dynamics after alloHSCT and by exploring potential sex-specific differences.

## 2 Materials and methods

In this retrospective study, we analyzed clinical records of 464 consecutive patients undergoing alloHSCT between 01/2012 and 08/2017 at the Department of Medicine I, University Hospital of Cologne, Germany. Patients aged ≥18 years with hematologic diseases and available pre-conditioning BMI were included and followed for survival and transplant-related toxicities until day+100 post-transplant. Underweight patients (BMI <18.5 kg/m²) were excluded due to limited numbers, resulting in a final cohort of 442 patients. For additional analyses, a transplant-homogenized cohort was defined, restricted to reduced-intensity conditioning (RIC), peripheral hematopoietic stem cell grafts, and full donor-recipient matching (matched related donor (MRD) or matched unrelated donor (MUD)). The study was conducted in accordance with the Declaration of Helsinki and approved (waiver) by the institutional review board (University of Cologne, Cologne, Germany).

### 2.1 Measurement of nutrition-associated parameters

BMI was assessed at initial diagnosis and pre-transplant according to World Health Organization (WHO) criteria (18). Blood cell counts and inflammatory markers were recorded at baseline (day+0, preTx) and on day+30 (d30). The mGPS was applied to define immunonutritional risk groups according to Proctor et al. (12): patients with normal CRP were assigned a score of 0; patients with elevated CRP (≥10 mg/L) and normal albumin received a score of 1; and patients with both elevated CRP (≥10 mg/L) and low albumin (<35 g/L) were assigned a score of 2. To assess dynamic changes in the immunonutritional status, increases in mGPS between baseline and day+30 (0→1, 0→2, 1→2) were differentiated (Δ^30^mGPS). Measurements were performed according to routine clinical laboratory standards at the University Hospital of Cologne.

### 2.2 Endpoints

OS, NRM and clinically relevant aGvHD (grade ≥2), were defined as endpoints. OS was defined as time from transplantation to death from any cause, with patients censored at last follow-up if alive. NRM was defined as time from transplantation to death from any cause other than relapse, with patients censored at relapse, progression, or last follow-up in the absence of an event. aGvHD grading within the first 100 days post-transplant was performed according to international standards (NIH criteria) (19). Outcomes were analyzed according to preTx mGPS, d30 mGPS and Δ^30^mGPS in the whole cohort as well as in sex-specific subgroups. Additionally, analyses were repeated in a transplant-homogenized cohort defined by disease and transplant setting. Based on the availability of mGPS data and the respective endpoints, numbers of patients included in the respective analyses varied, and details on the numbers are given in the appropriate figure legends.

### 2.3 Statistical analysis

Patient and transplant characteristics were compared between mGPS classes using the chi-square test for categorical variables and the Kolmogorov-Smirnov test for non-parametric continuous variables. Normality of continuous variables was assessed using the Kolmogorov-Smirnov test and Q-Q-Plots. Continuous variables were reported as median with range, and categorical variables as counts with percentages.

To compare OS, NRM and aGvHD rates between mGPS groups, we used the Kaplan-Meier method and log-rank test for survival analyses (OS and NRM), and the cumulative incidence of aGvHD was estimated by 1 – survival probability. Hazard ratios (HR) with corresponding 95% confidence intervals (CI) were calculated to estimate effect sizes in pairwise comparisons using mGPS 0 as reference.

Univariable Cox regression analyses were performed for OS, NRM, and aGvHD, including age, sex, conditioning regimen, graft source, donor matching type, HCT-CI, preTx mGPS, Δ^30^mGPS and aGvHD occurring before d+30 as parameters. Variables that were significant in univariable analyses or considered relevant based on clinical knowledge were entered in a multivariable Cox regression model. To quantify the added importance of preTx mGPS and Δ^30^mGPS, respectively, compared to a nested model containing only the HCT-CI and all covariates, a p-value based on the likelihood ratio test was calculated. Multicollinearity was evaluated using the variance inflation factor (VIF), with VIF <5 considered acceptable. Statistical analysis was performed using GraphPad Prism v10.1.1 and IBM SPSS Statistics v32, with a significance level of p ≤ 0.05.

## 3 Results

### 3.1 Description of the cohort and transplantation setting

**Figure 1:**
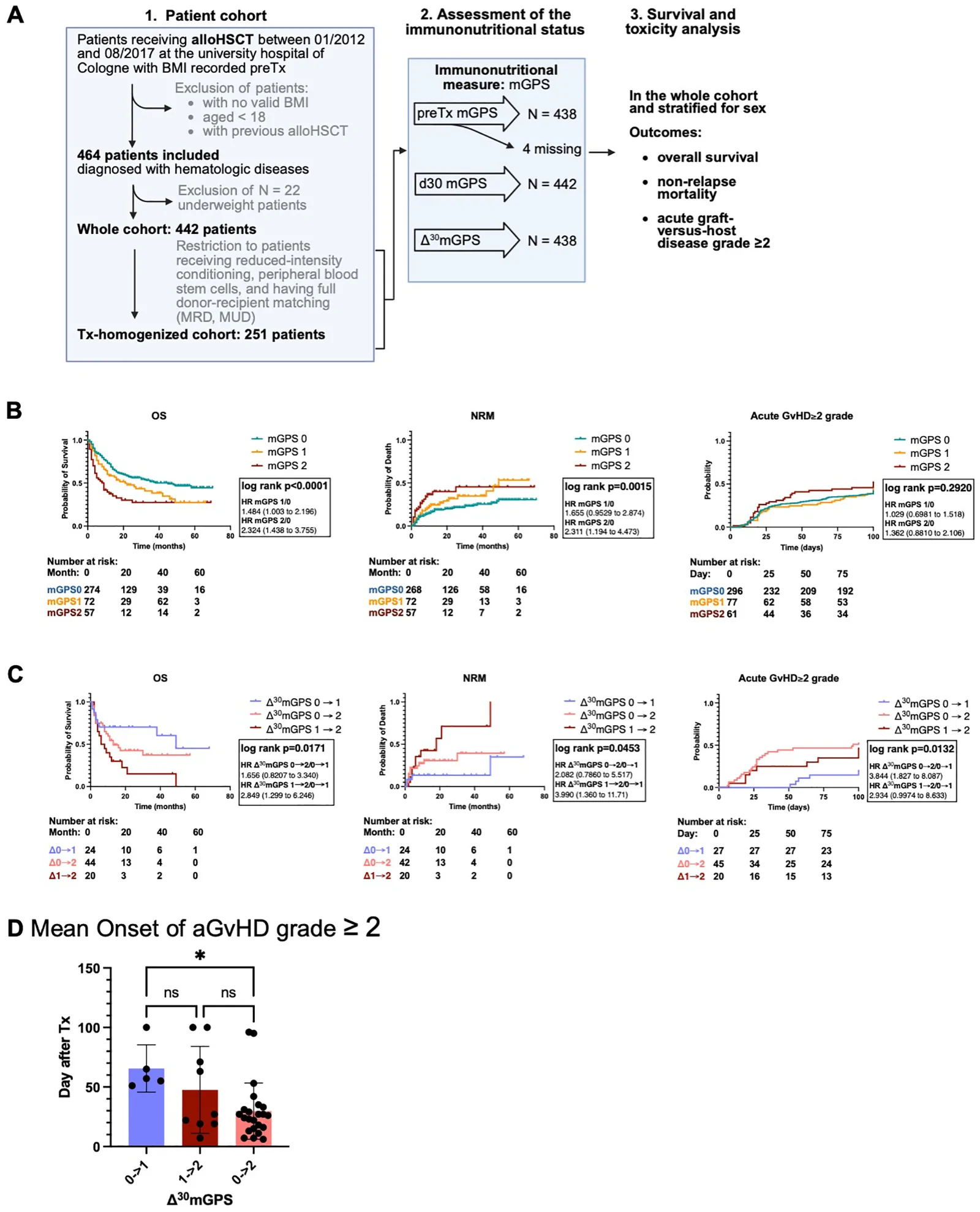
Pre-transplant mGPS and its early post-transplant dynamics are associated with OS, NRM and aGvHD to a different extend. **(A)** Schematic outline and PRISMA diagram of the study. The whole cohort comprised 442 patients. Based on availability of mGPS data and the respective outcomes, patient numbers varied. **(B)** Based on preTx mGPS, Kaplan-Meier plots were generated for the whole cohort addressing OS (n = 403), NRM (n = 397) and the cumulative incidence for aGvHD grade ≥2 (n = 434). **(C)** Based on Δ^30^mGPS, similar analyses were performed: OS (n = 88), NRM (n = 86) and aGvHD grade ≥2 (n = 92). **(D)** Scatter plots show the mean onset (days after Tx) of aGvHD grade ≥2 according to Δ^30^mGPS. Statistical analyses were performed with the log-rank test for survival curves and cumulative incidence of aGvHD. The Kruskal-Wallis test was employed to test differences in aGvHD onset. Statistical significance is indicated with an asterix (*p<0.05).

Survival and toxicity outcomes were assessed in 442 patients undergoing alloHSCT for myeloid neoplasms (63%), high-grade non-Hodgkin lymphoma/acute lymphoblastic leukemia (19%), indolent non-Hodgkin lymphoma (13%), and Hodgkin lymphoma (3%) (**Table 1**; **Figure 1A**). Underweight patients (n = 22) were excluded due to limited numbers and potential confounding by comorbidities, inflammation, and metabolic alterations (20). AlloHSCT was predominantly performed using RIC (77%) and peripheral blood stem cells (97%). The median number of transplanted CD34+ cells was 6.7 x 10^6^/kg (range 1.1**–**21.9). HLA-based (10 items) donor-recipient matching included 51% MRD, 23% MUD, 21% mismatched (8/10 or 9/10), and 5% haploidentical donors.

**Table 1:** Baseline patient characteristics and details of the transplantation setting in the whole cohort and sex-specific subgroups.

Variable		Whole cohort (N = 442; 100%)	Females (N = 187; 42%)	Males (N = 255; 58%)	P-value
Age, median (range) (years)		57 (18–76)	57 (18–75)	57 (19–76)	0.22
Diagnosis, n (%)					0.20
Hodgkin Lymphoma		11 (2)	6 (3)	5 (2)	
Myeloid Neoplasms^1^		280 (63)	121 (65)	159 (62)	
High-grade Non-Hodgkin Lymphoma/Acute Lymphoblastic Leukemia^2^		84 (19)	39 (21)	45 (18)	
Indolent Non-Hodgkin Lymphoma^3^		59 (13)	17 (9)	42 (16)	
Others^4^		8 (2)	4 (2)	4 (2)	
Karnofsky Index (%), median (range)		90 (50–100)	90 (60–100)	100 (50–100)	0.87
HCT-CI Score, median (range)		2 (0–10)	3 (0–10)	2 (0–10)	0.62
PreTx mGPS, n (%)					0.12
0		297 (67)	126 (67)	171 (67)	
1		77 (17)	32 (17)	45 (18)	
2		64 (14)	25 (13)	39 (15)	
Missing		4 (1)	4 (2)	0 (0)	
Transplantation setting					
Conditioning, n (%)					0.85
Myeloablative Conditioning (MAC)		88 (20)	36 (19)	52 (20)	
Reduced intensity conditioning (RIC)		342 (77)	145 (78)	197 (77)	
Mini Conditioning		9 (2)	4 (2)	5 (2)	
Unknown		3 (1)	2 (1)	1 (0)	
Method of stem cell collection, n (%)					0.48
Bone Marrow		13 (3)	6 (7)	7 (3)	
Peripheral Blood Stem Cells (pBSC)		428 (97)	180 (248)	248 (97)	
Unknown		1 (0)	1 (1)	0 (0)	
Graft (x10^6^/kg BW), median (range)		6.7 (1.1–21.9)	6.9 (2.8–20.0)	6.4 (1.1–21–6)	0.26
Donors, n (%)					0.18
Matched Related Donor (MRD)		102 (23)	34 (18)	68 (27)	
Matched Unrelated Donor (MUD)		225 (51)	100 (53)	125 (49)	
Mismatched Donor (8/10 and 9/10)		94 (21)	42 (22)	52 (20)	
Haploidentical		21 (5)	11 (6)	10 (4)	
Recipient/Donor CMV status					0.78
R+/D+		187 (42)	80 (43)	107 (42)	
R+/D-		74 (17)	32 (17)	42 (16)	
R-/D+		40 (9)	20 (11)	20 (8)	
R-/D-		139 (31)	54 (29)	85 (33)	
Unknown		2 (0)	1 (1)	1 (0)	

^1^ acute myeloic lymphoma, myelodysplastic syndrome, myeloproliferative disease; ^2^aggressive non-Hodgkin lymphoma, acute lymphatic leukemia, lymphoblastic lymphoma; ^3^chronic lymphatic leukemia; ^4^severe aplastic anemia, hemoglobin disorder.

Of the 94 mismatched donors, 3 are related and 91 are unrelated.

Baseline transplant characteristics did not differ significantly by sex or mGPS group (**Table 1**; **Supplementary Table 1**). Median age was 57 years (range 18**–**76) and the whole cohort comprised 42% females and 58% males. PreTx mGPS was 0 in 297 patients (67%), 1 in 77 patients (17%), and 2 in 64 patients (14%); data on CRP and albumin were missing in 4 cases. Most patients had a high Karnofsky Index (median 90%, range 50**–**100) and an intermediate risk for unfavourable outcomes due to comorbidity (median HCT-CI 2, range 0**–**10) (6). Risk scores were balanced between sexes.

### 3.2 PreTx mGPS and its dynamic changes within 30 days post-transplant correlate significantly with OS, NRM, and aGvHD incidence.

In the whole cohort, median OS differed significantly across preTx mGPS groups: 45 months for mGPS 0, 21 months for mGPS 1, and 9 months for mGPS 2 (p<0.0001). The mGPS stratified patients into three ordered risk groups, with HRs of 1.48 (95% CI 1.00**–**2.20) for mGPS 1 and 2.32 (95% CI 1.44**–**3.76) for mGPS 2 versus mGPS 0. The 5-year NRM was 36.4% overall and increased with higher mGPS (HR_mGPS1/0_, 1.66; 95% CI 0.95**–**2.87; HR_mGPS2/0_, 2.31; 95% CI 1.19**–**4.47; p=0.002). Day+100 aGvHD incidence was 42.5%. PreTx mGPS was not associated with aGvHD (HR_mGPS1/0_, 1.03; 95% CI 0.70**–**1.52; HR_mGPS2/0_, 1.36; 95% CI 0.88**–**2.11; p=0.29), although a trend toward lower incidence for mGPS 1 was observed (**Figure 1B**). To check for major biases in the whole cohort we additionally analyzed a transplant-homogenized cohort based on the factors RIC, peripheral hematopoietic stem cell grafts, and full donor-recipient matching (MRD or MUD). Here we found that for all three outcomes the curve patterns and the HRs were comparable to the whole cohort (**Supplementary Figure 1A**).

To assess whether individual mGPS category changes occur within the first 30 days post-transplant, mGPS was re-evaluated at day+30 and categorized relative to baseline (Δ^30^mGPS). Δ^30^mGPS dynamics were significantly associated with all outcomes, including aGvHD (OS p=0.02; NRM p=0.05; aGvHD p=0.01). For OS and NRM, a shift from 1→2 conferred the worst prognosis compared with 0→1 (OS HR 2.85, 95% CI 1.30**–**6.25; NRM HR 3.99, 95% CI 1.36**–**11.71), whereas for aGvHD, 0→2 was most adverse (HR 3.84, 95% CI 1.83**–**8.09) (**Figure 1C**). D30 mGPS alone showed similar associations (**Supplementary Figure 1B**).

Given the lack of a pre-transplant association of the mGPS with aGvHD in contrast to its post-transplant performance, we further explored its relationship with aGvHD. aGvHD onset occurred significantly earlier in patients with Δ^30^mGPS 0→2 (mean onset day+30) than in those with 0→1 (day+66) (**Figure 1D**). No such association was observed for preTx mGPS (**Supplementary Figure 1C**). Maximum aGvHD grade ≥2 did not differ meaningfully between Δ^30^mGPS groups (**Supplementary Figure 1D**). Diverging Kaplan–Meier curves of Δ^30^mGPS and d30 mGPS within the first 30 days, followed by parallel trajectories thereafter, raised the question of whether post-transplant mGPS could serve as a useful prospective marker of aGvHD incidence or rather reflects early aGvHD, given that a substantial proportion of aGvHD cases occur within the first 30 days. Accordingly, censoring for events before day+30 preserved the association of Δ^30^mGPS with OS and NRM but abolished it for aGvHD (OS p=0.003; NRM p=0.005; aGvHD p=0.81) (**Supplementary Figures 1E, F**).

### 3.3 Multivariable analysis indicates independent prognostic value of the mGPS and its dynamics in addition to the HCT-CI.

Multivariable models included preTx mGPS and Δ^30^mGPS, each adjusted for age, sex, conditioning regimen, graft source, donor matching type and HCT-CI. For the analysis of OS and NRM according to Δ^30^mGPS, aGvHD before day+30 was included as a separate risk factor to adjust for a potential association of Δ^30^mGPS with early development of aGvHD. For the analyses of aGvHD incidence according to Δ^30^mGPS, patients who developed aGvHD or died before day+30 were censored. As shown in **Tables 2**, **3**, both univariable and multivariable Cox regression analyses confirmed significant, graded associations of preTx mGPS and Δ^30^mGPS with OS and NRM, whereas no statistically significant associations were observed for aGvHD. For OS and NRM, Δ^30^mGPS 1→2 was the most adverse (HR_OS_ 3.40, p<0.001; HR_NRM_ 3.38, p=0.001) using Δ^30^mGPS 0→0 as the reference. This was followed by Δ^30^mGPS 0→2 (HR_OS_ 1.72, p=0.03; HR_NRM_ 2.00, p=0.04), whereas Δ^30^mGPS 0→1 was not associated with survival (HR_OS_ 1.48, p=0.26; HR_NRM_ 1.42, p=0.48). No significant association of preTx mGPS or Δ^30^mGPS with aGvHD incidence was observed, although Δ^30^mGPS 0→1 showed a non-significant trend toward a lower hazard of aGvHD (HR 0.54, p=0.23) (**Table 3**). Likelihood ratio testing demonstrated that models including preTx mGPS or Δ^30^mGPS provided additional prognostic value for OS (preTx mGPS: p<0.001; Δ^30^mGPS: p<0.001) and NRM (preTx mGPS: p=0.03; Δ^30^mGPS: p=0.01) compared with the nested model including HCT-CI and the other covariates alone (**Supplementary Table 2**).

**Table 2:** Univariable Cox regression analysis of OS, NRM and aGvHD grade ≥2.

Variable		OS		NRM		Acute GvHD grade ≥2	
		N	HR (95% CI) p-value	N	HR (95% CI) p-value	N	HR (95% CI) p-value
Age		412	1.01 (1.00–1.02) p=0.04	406	1.02 (1.01–1.04) p=0.01	438	0.99 (0.98–1.00) p=0.24
Sex							
Female (Ref.)		180		179		186	
Male		232	0.86 (0.65–1.12) p=0.26	227	1.01 (0.69–1.48) p=0.97	252	0.99 (0.74–1.33) p=0.95
Conditioning							
Myeloablative (MAC) (Ref.)		81		80		88	
Reduced Intensity (RIC)		320	1.15 (0.81–1.62) p=0.44	315	1.20 (0.73–1.98) p=0.47	338	0.95 (0.66–1.36) p=0.77
Mini Conditioning		8	3.17 (1.34–7.49) p=0.01	8	2.76 (0.81–9.37) p=0.10	9	1.03 (0.37–2.87) p=0.96
Graft source							
pBSC (Ref.)		401		395		424	
Bone Marrow		10	1.39 (0.66–2.96) p=0.39	10	1.50 (0.55–4.08) p=0.43	13	1.58 (0.74–3.36) p=0.24
Donor matching type							
MRD (Ref.)		96		95		102	
MUD		211	1.24 (0.87–1.77) p=0.25	208	1.41 (0.83–2.41) p=0.21	223	1.06 (0.74–1.54) p=0.75
Mismatched (8/10 and 9/10)		87	1.95 (1.30–2.93) p=0.001	86	2.49 (1.39–4.46) p=0.002	94	1.45 (0.95–2.21) p=0.08
Haploidentical		18	1.20 (0.54–2.66) p=0.66	17	2.16 (0.80–5.81) p=0.13	19	1.04 (0.49–2.22) p=0.92
HCT-CI							
Low (0–2) (Ref.)		205		200		220	
High (≥3)		185	1.25 (0.94–1.66) p=0.12	184	1.62 (1.09–2.41) p=0.02	197	1.05 (0.78–1.41) p=0.76
PreTx mGPS							
0 (Ref.)		275		269		296	
1		73	1.50 (1.06–2.12) p=0.02	73	1.68 (1.05–2.71) p=0.03	77	1.03 (0.70–1.51) p=0.88
2		60	2.52 (1.77–3.60) p<0.001	60	2.73 (1.68–4.44) p<0.001	61	1.36 (0.92–2.03) p=0.12
Δ^30^mGPS							
0→0 (Ref.)		206		202		170*	
0→1		25	1.17 (0.61–2.26) p=0.63	25	1.22 (0.48–3.10) p=0.67	18*	0.48 (0.17–1.32) p=0.16
0→2		44	1.85 (1.17–2.90) p=0.01	42	2.18 (1.16–4.10) p=0.02	33*	1.37 (0.79–2.36) p=0.27
1→2		20	3.49 (2.07–5.89) p<0.001	20	3.95 (1.95–7.95) p<0.001	18*	1.10 (0.53–2.29) p=0.80
aGvHD before d+30							
No (Ref.)		310		306			
Yes		102	1.27 (0.94–1.713) p=0.13	100	1.54 (1.03–2.32) p=0.04		

The whole cohort comprised 442 patients. Based on availability of data on the variable and the respective outcomes, patient numbers varied.

*For the analysis of aGvHD and Δ^30^mGPS, it was censored for aGvHD cases occurring before d+30 or dying before d+30.

**Table 3:** Multivariable Cox regression analysis of OS, NRM and aGvHD grade ≥2.

Variable		OS		NRM		Acute GvHD grade ≥2	
		N	HR (95% CI) p-value	N	HR (95% CI) p-value	N	HR (95% CI) p-value
PreTx mGPS^a^							
0 (Ref.)		260		254		281	
1		72	1.52 (1.06–2.17) p=0.02	72	1.56 (0.95–2.56) p=0.08	76	0.99 (0.67–1.47) p=0.96
2		54	2.34 (1.57–3.51) p<0.001	54	2.07 (1.19–3.61) p=0.01	56	1.27 (0.82–1.97) p=0.28
Δ^30^mGPS^b^							
0→0 (Ref.)		194		190		160*	
0→1		24	1.48 (0.75–2.91) p=0.26	24	1.42 (0.54–3.74) p=0.48	17*	0.54 (0.19–1.48) p=0.23
0→2		42	1.72(1.05–2.81) p=0.03	40	2.00 (1.03–3.91) p=0.04	32*	1.45 (0.82–2.55) p=0.20
1→2		20	3.40 (1.97–5.87) p<0.001	20	3.38 (1.62–7.07) p=0.001	18*	1.10 (0.51–2.35) p=0.81

The whole cohort comprised 442 patients. Based on availability of data on the variable and the respective outcomes, patient numbers varied.

*For the analysis of aGvHD and Δ^30^mGPS, it was censored for aGvHD cases occurring before d+30 or dying before d+30.

^a^Covariates for the analyses were: age, sex, conditioning regimen (MAC vs others), graft source (pBSC vs. bone marrow), donor matching type (MRD vs others) and HCT-CI (low vs high).

^b^ Covariates for the analyses were: age, sex, conditioning regimen (MAC vs others), graft source (pBSC vs. bone marrow), donor matching type (MRD vs others), HCT-CI (low vs high) and for OS and NRM additionally aGvHD before d+30 (No vs Yes).

### 3.4 Sex-specific performance of the preTx mGPS

**Figure 2:**
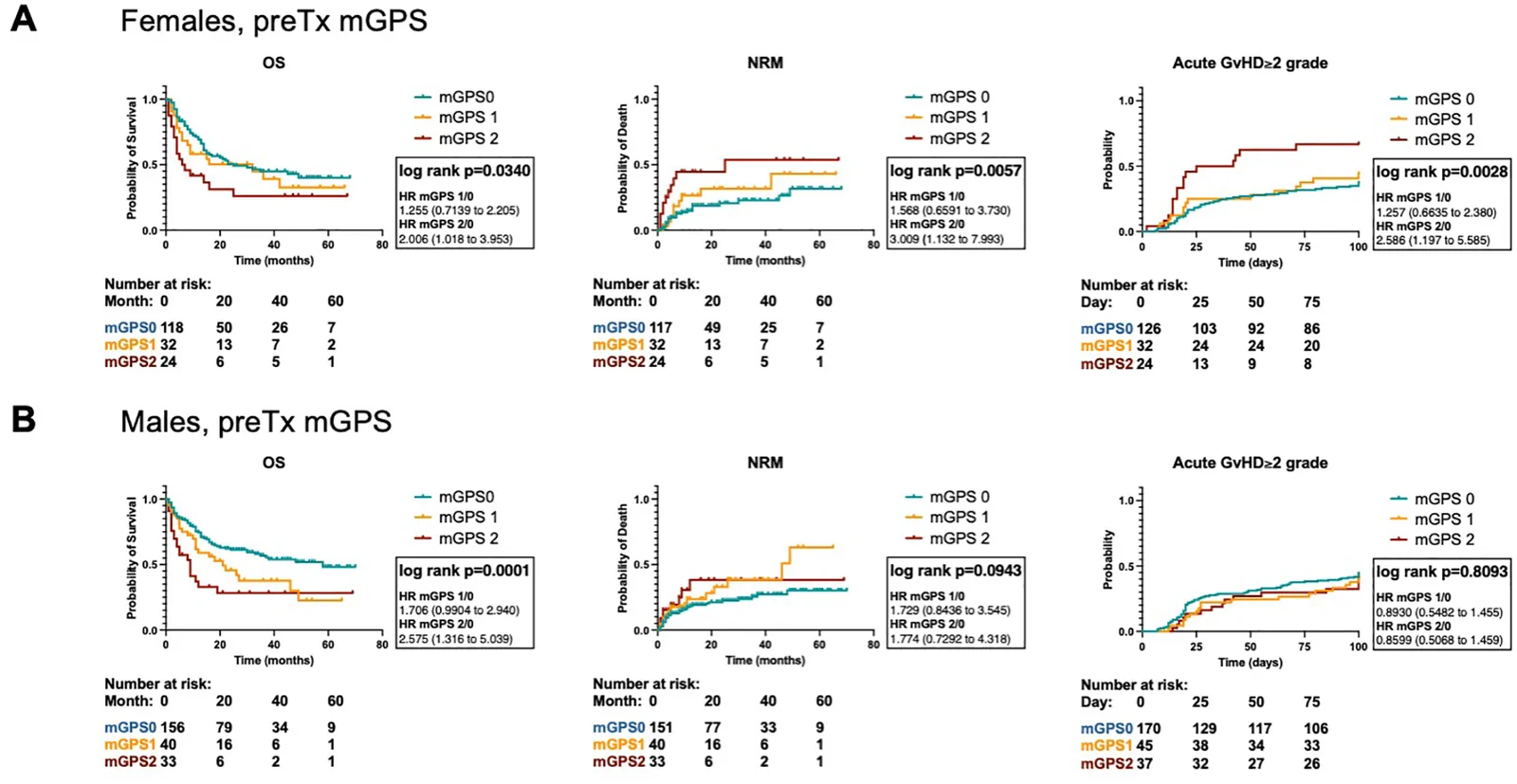
Sex-specific associations of the preTx mGPS according to OS, NRM and aGVHD. The whole cohort comprised 187 females and 255 males. Based on availability of mGPS data and the respective outcomes, patient numbers varied. **(A)** For all females, Kaplan-Meier plots were generated according to preTx mGPS addressing OS (n = 174), NRM (n = 173) and the cumulative incidence for aGvHD grade ≥2 (n = 182). **(B)** For all male patients, similar analyses were performed: OS (n = 229), NRM (n = 224) and aGvHD grade ≥2 (n = 252). Statistical analyses were performed with the log-rank test for survival curves and cumulative incidence of aGvHD.

To understand the impact of sex-specific differences on mGPS performance, survival and toxicity outcomes were analyzed separately for females and males, shown in **Figure 2**; **Supplementary Figures 2B, C**. Median OS differed significantly across preTx mGPS groups in both females and males (p_females_=0.03; p_males_=0.0001) with the poorest OS observed in mGPS 2 (HR_females_ 2.01, 95% CI 1.02**–**3.95; HR_males_ 2.58, 95% CI 1.32**–**5.04). In females, preTx mGPS was also significantly associated with NRM, whereas this trend did not reach statistical significance in males (p_females_=0.01; p_males_=0.09). Notably, preTx mGPS was associated with aGvHD incidence in females (p_females_=0.003): patients with mGPS 2 had a higher risk of developing aGvHD grade ≥ 2 compared with mGPS 0 (HR 2.59, 95% CI 1.20–5.59). No such association was observed in males (p_males_=0.81; HR_mGPS2/0_ 0.86, 95% CI 0.51**–**1.46) (**Figures 2A, B**).

### 3.5 Sex-specific performance of early post-transplant mGPS changes

**Figure 3:**
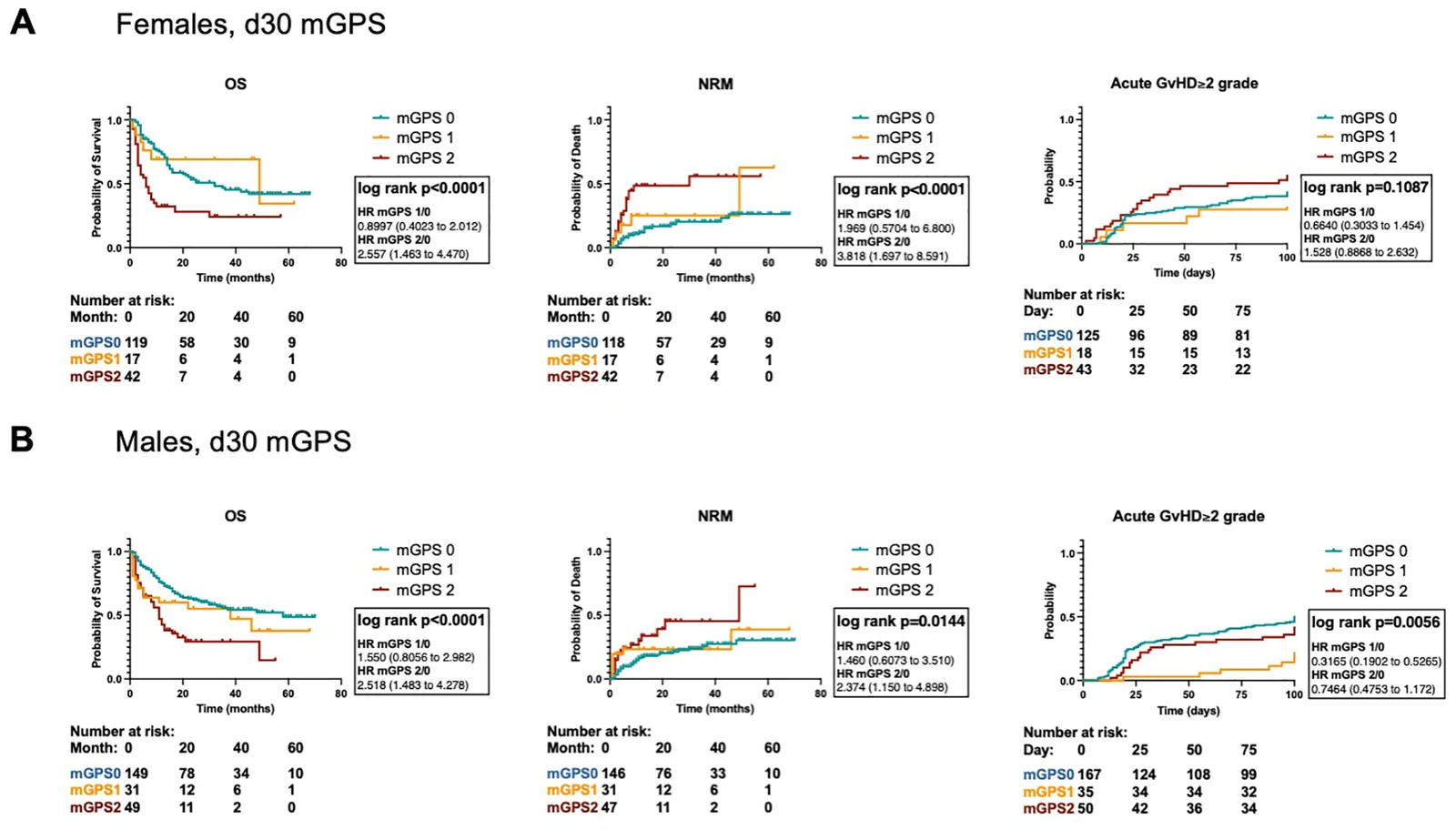
Sex-specific performance of the mGPS and its early dynamics post-transplant. The whole cohort comprised 187 females and 255 males. Based on availability of mGPS data and the respective outcomes, patient numbers varied. **(A)** For all female patients, similar analyses according to d30 mGPS were performed: OS (n = 178), NRM (n = 177) and aGvHD grade ≥2 (n = 186). **(B)** For all male patients, similar analyses according to d30 mGPS were performed: OS (n = 229), NRM (n = 224) and aGvHD grade ≥2 (n = 252). Statistical analyses were performed with the log-rank test for survival curves and cumulative incidence of aGvHD.

Sex-specific analyses of d30 mGPS confirmed a strong association of the mGPS in its post-transplant course with OS and NRM in both sexes (all p ≤ 0.01). Most adverse for OS and NRM was d30 mGPS 2 (OS_females_ HR 2.56, 95% CI 1.46**–**4.47; NRM_females_ HR 3.82, 95% CI 1.70**–**8.59; OS_males_ HR 2.52, 95% CI 1.48**–**4.28; NRM_males_ HR 2.37, 95% CI 1.15**–**4.90), without evidence of sex-specific differences. For aGvHD, however, a significant association was observed only in males (p_females_=0.11; p_males_=0.01): mGPS 1 was associated with significantly lower aGvHD incidence compared to mGPS 0 (HR 0.32, 95% CI 0.19–0.53), whereas aGvHD incidence was similarly high in mGPS 2 and mGPS 0 (**Figures 3A, B**).

The Δ^30^mGPS was not significantly associated with OS, NRM, or aGvHD in either females or males. However, Kaplan-Meier curves and HRs suggested patterns consistent with the whole cohort, but subgroup sizes were limited (females: n = 39–41; males: n = 47–51). No clear sex-specific effects of Δ^30^mGPS were observed (**Supplementary Figures 3A–D**).

## 4 Discussion

While the mGPS is a validated prognostic marker in solid cancers, its role in alloHSCT remains insufficiently defined. Previous studies have primarily focused on single inflammatory or nutritional biomarkers, which may not adequately capture the complex biological processes underlying post-transplant outcomes. To our knowledge, this is the first study to evaluate the combined immunonutritional mGPS both pre-transplant and longitudinally during the early post-transplant period. We additionally explored sex-specific patterns given known differences in immune response and metabolism between males and females (21, 22). Overall, our data indicate that the mGPS is associated with OS and NRM, particularly in its early post-transplant dynamics, whereas findings regarding aGvHD were less consistent.

The associations of preTx mGPS with OS and NRM are consistent with previous reports (3, 8). However, no significant association with aGvHD was observed. Evidence on these relationships remains limited, particularly regarding aGvHD, and is largely restricted to studies of single immunonutritional biomarkers such as CRP or albumin, which – together with heterogeneous definitions – limits comparability across studies. In line with our findings, several studies report no association between baseline CRP and aGvHD (23, 24). However, Fuji et al. reported associations with both NRM and aGvHD (22).

A key novel aspect of our study is the extension of mGPS assessment to the post-transplant period, capturing early dynamics and underscoring the importance of immunonutritional status beyond transplantation. Both d30 mGPS and Δ^30^mGPS were strongly associated with OS and NRM. Although d30 mGPS and Δ^30^mGPS were associated with aGvHD in the primary analysis, this association disappeared after censoring events before day+30. Importantly, not only absolute post-transplant values but particularly dynamic changes may refine risk stratification. Δ^30^mGPS integrates pre-existing inflammatory status and early post-transplant evolution, thereby providing more granular prognostic information. This extends previous findings by Schaffrath et al., who reported a prognostic value of pre- and post-transplant hypoalbuminemia for NRM but a limited value of post-transplant dynamics (2). In contrast, our data suggest that dynamic assessment might be clinically meaningful. Supporting this, Rashidi et al. reported an association of post-transplant albumin decline with severe aGvHD and Artz et al. found no association between pre-transplant albumin and aGvHD (25, 26).

Our findings suggest that early post-transplant mGPS changes may reflect ongoing or resolving inflammatory processes, including early aGvHD, rather than predict subsequent aGvHD. Given the limited sample size and the complex immunonutritional processes between day+0 and day+30, no firm conclusions regarding the predictive value of Δ^30^mGPS can be drawn. Future studies with serial mGPS assessments may help to clarify the prognostic relevance of early post-transplant mGPS dynamics. This interpretation aligns with Miyazaki et al., who concluded that the CRP-to-albumin ratio might reflect transplant-associated physiological impairment or the underlying hematologic disease itself rather than being a pure predictive marker (8). Similarly, Chung et al. demonstrated an association of high post-transplant GPS with steroid resistance and NRM in alloHSCT patients (27), suggesting that steroid refractoriness may contribute to the association between mGPS dynamics and aGvHD. Interestingly, we found mGPS 1 at day+30 being associated with a 46% lower risk for aGvHD than mGPS 0, while individuals with mGPS 2 had the highest risk to develop clinically relevant aGvHD. This is counterintuitive at first site, since mGPS 1 is characterized by increased inflammation (elevated CRP), while a hyper-catabolic state (low albumin) is absent. In lack of experimental data, we can only hypothesize on potential underlying mechanisms. Rather than low-grade inflammation itself being protective, published data suggest that controlled inflammatory signaling coupled with active resolution mechanisms, e.g. anti-inflammatory cytokine secretion such as interleukin-10 or interleukin-6, could limit aGvHD (28). Here, CRP levels would be still elevated and might reflect resolving inflammation. On the other hand, low serum albumin levels have been numerously shown as a strong predictor of poor prognosis in a wide range of critical illnesses including aGvHD (27) and our data on mGPS 2 as the highest risk group are in line with these observations.

Given increasing evidence that sex-related differences in body composition, inflammatory responses, and metabolic regulation may influence outcomes of hematological therapies (17, 29–31), we explored sex-specific differences in the prognostic relevance of mGPS in alloHSCT. Sex-specific analyses suggested distinct patterns, particularly regarding OS and NRM. In both sexes, early post-transplant mGPS dynamics were more strongly associated with OS and NRM than preTx mGPS, with somewhat stronger associations for NRM in females. In our cohort, aGvHD incidence and median time to onset were similar between females and males (**Supplementary Figures 2A–C**). Although mGPS showed potential sex-related patterns regarding aGvHD, these findings were inconsistent and limited by small subgroup sizes. Nevertheless, they raise the hypothesis that sex-related biological differences may modify the relationship between immunonutritional status and post-transplant outcomes, suggesting that sex may need to be considered when interpreting immunonutritional scores. The underlying mechanisms remain poorly understood but may involve differences in inflammatory regulation, body composition, hormonal status, microbiota composition, and metabolic reserve (10, 32–36). In addition, donor-recipient sex combinations have been associated with transplant outcomes, with sex mismatch potentially contributing to aGvHD development (37–39), further supporting the relevance of sex-related biological factors.

Limitations of this study include the retrospective single-center design and limited sample size, especially for subgroup analyses. This restricts our ability to draw firm conclusions, particularly regarding the sex-specific Δ^30^mGPS analyses. Therefore, these findings should be considered exploratory and require validation in larger prospective cohorts. Furthermore, day+30 mGPS measurements may be influenced by complex and rapidly evolving post-transplant processes, limiting specificity, as both albumin and CRP reflect multiple conditions including infectious complications beyond nutrition and chronic inflammation. The exclusion of underweight patients may limit the generalizability of our findings. Importantly, undernutrition represents only one aspect of malnutrition, which encompasses heterogeneous phenotypes such as cachexia, sarcopenia, sarcopenic obesity, and obesity. Future studies should capture potential confounding conditions, such as infectious complications, and include a broader spectrum of nutritional phenotypes.

Strengths include the integration of two biomarkers reflecting distinct aspects of metabolic and inflammatory processes into a single variable, evaluated in a relatively large cohort encompassing heterogeneous transplantation settings. External validation in larger multicenter prospective cohorts is warranted. Emerging approaches such as ML may help integrate multidimensional immunonutritional data beyond established scores such as the mGPS, potentially allowing more refined risk stratification and deeper insights into mechanisms underlying post-transplant outcomes (14–16).

In conclusion, the mGPS represents a valuable prognostic marker in alloHSCT, particularly in its early post-transplant dynamics. The observed sex-specific patterns emphasize the importance of considering sex differences in prognostic interpretation. These findings support the role of immunonutritional status in individualized risk stratification and early therapeutic decision-making.

## Data Availability

The raw data supporting the conclusions of this article will be made available by the authors upon reasonable request.
