# Supplementary material for: Early and sex-specific dynamic changes of the modified Glasgow Prognostic Score correlate with survival and immunotoxicities in patients undergoing allogeneic hematopoietic stem cell transplantation
Source: Front Immunol. 2026 Aug 17;17:1894926. doi: 10.3389/fimmu.2026.1894926 (PMC13526978; doi:10.3389/fimmu.2026.1894926)
Supplement: Supplementary file 1 [file SupplementaryFile1.pdf]

Supplementary Table S1. Baseline patient characteristics and details of the transplantation setting in the whole cohort and according to mGPS subgroups.

Variable	Whole cohort (N=442; 100%)	PreTx mGPS 0 (N=297; 67%)	PreTx mGPS 1 (N=77; 17%)	PreTx mGPS 2 (N=64; 14%)	p-value
Age, median (range) (years)	57 (18–76)	57 (18–76)	58 (19–75)	59 (22–75)	0.10
Gender, n (%)					0.88
female	187 (42)	126 (42)	32 (42)	25 (39)	
male	255 (58)	171 (58)	45 (58)	39 (61)	
Diagnosis, n (%)					0.03
Hodgkin Lymphoma	11 (2)	6 (2)	4 (5)	0 (0)	
Myeloic Neoplasms[1]	280 (63)	186 (63)	48 (62)	44 (69)	
High-grade Non-Hodgkin Lymphoma/ Acute Lymphoblastic Leukemia[2]	84 (19)	64 (22)	13 (17)	7 (11)	
Indolent Non-Hodgkin Lymphoma[3]	59 (13)	39 (13)	8 (10)	11 (17)	
Others[4]	8 (2)	2 (1)	4 (5)	2 (3)	
Karnofsky Index (%), median (range)	90 (50–100)	100 (60–100)	90 (50–100)	90 (50–100)	<0.0001
HCT-CI Score, median (range)	2 (0–10)	2 (0–9)	3 (0–9)	3.5 (0–10)	<0.0001
**Transplantation setting**					
Conditioning, n (%)					0.29
Myeloablative Conditioning (MAC)	88 (20)	66 (22)	16 (21)	6 (9)	
Reduced intensity conditioning (RIC)	342 (77)	224 (75)	59 (77)	55 (86)	
Mini Conditioning	9 (2)	6 (2)	1 (1)	2 (3)	
Unknown	3 (1)	1 (0)	1 (1)	1 (2)	
Method of Stem Cell Collection, n (%)					0.35
Bone Marrow	13 (3)	5 (2)	5 (7)	3 (5)	
Peripheral Blood Stem Cells (pBSC)	428 (97)	292 (98)	72 (94)	61 (95)	
Unknown	1 (0)	0 (0)	0 (0)	0 (0)	
Graft (x10^6/kg BW), median (range)	6.7 (1.1–21.9)	6.5 (1.1–20.7)	7.6 (1.7–20.0)	7.2 (1.6–21.9)	0.31
Donors, n (%)					0.29
Matched Related Donor (MRD)	102 (23)	70 (24)	13 (17)	18 (28)	
Matched Unrelated Donor (MUD)	225 (51)	153 (52)	43 (56)	28 (44)	
Mismatched Donor (8/10 and 9/10)	94 (21)	60 (20)	20 (26)	13 (20)	
Haploidentical	21 (5)	14 (5)	1 (1)	5 (8)	
Recipient/Donor CMV status					0.56
R+/D+	187 (42)	127 (43)	31 (40)	27 (42)	
R+/D-	74 (17)	47 (16)	16 (21)	11 (17)	
R-/D+	40 (9)	30 (10)	5 (6)	4 (6)	
R-/D-	139 (31)	93 (31)	24 (31)	21 (33)	
Unknown	2 (0)	0 (0)	1 (1)	1 (2)	

[1] acute myeloic lymphoma, myelodysplastic syndrome, myeloproliferative disease; [2] aggressive non-Hodgkin lymphoma, acute lymphatic leukemia, lymphoblastic lymphoma; [3] chronic lymphatic leukemia; [4] severe aplastic anemia, hemoglobin disorder.

Of the 94 mismatched donors, 3 are related and 91 are unrelated.

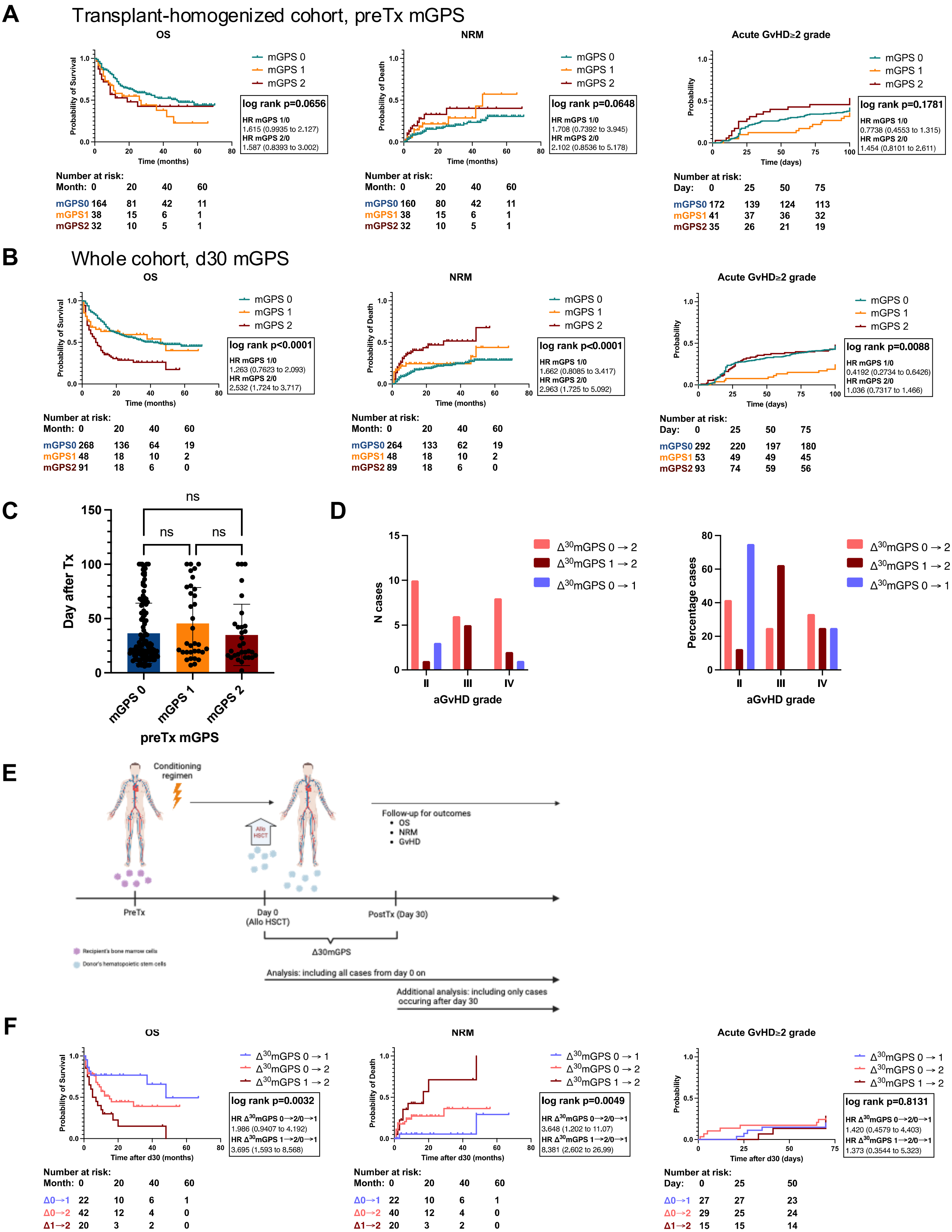

Supplementary Figure S1. (**A**) In the transplant-homogenized cohort (n=251) according to preTx mGPS, Kaplan-Meier plots were generated addressing OS (n = 234), NRM (n = 230) and the cumulative incidence for aGvHD grade ≥2 (n = 248). (**B**) In the whole cohort according to d30 mGPS, similar analyses were performed: OS (n = 407), NRM (n = 401) and aGvHD grade ≥ 2 (n = 438). In order to explore aGvHD more in detail, (**C**) the mean onset of an aGvHD grade ≥2 in days according to preTx mGPS was depicted and (**D**) the maximum aGvHD grade within Δ^{30}mGPS groups in totals and percentages was differentiated. (**E**) Explanatory figure for analyses timeline, i.e. additional analysis from day+30 on (Created in https://BioRender.com). (**F**) Survival and toxicity analysis as above from day+30 onwards according to Δ^{30}mGPS in the whole cohort: OS (n = 84), NRM (n = 82) and aGvHD grade ≥2 (n = 71).
Statistical analyses were performed with the log-rank test for survival curves and cumulative incidence of aGvHD. The Kruskal-Wallis test was employed to test differences in aGvHD onset.

Supplementary Table S2. LR test statistic comparing a model containing preTx mGPS or Δ[30]mGPS in addition to the nested model containing only the HCT-CI and all covariates.

Variable	OS	NRM	aGvHD grade ≥2
preTx mGPS[a]	χ^2 = 17.29, df = 2, p<0.001	χ^2 = 7.39, df = 2, p=0.03	χ^2 = 1.19, df = 2, p=0.55
Δ[30]mGPS[b]	χ^2 = 18.05, df = 3, p<0.001	χ^2 = 11.01, df = 3, p=0.01	χ^2 = 3.80, df = 3, p=0.28

The whole cohort comprised 442 patients. Based on availability of data on the variable and the respective outcomes, patient numbers varied.
* For the analysis of aGvHD and Δ[30]mGPS, it was censored for aGvHD cases occurring before d+30 or dying before d+30.
[a] Covariates for the analyses were: age, sex, conditioning regimen (MAC vs others), graft source (pBSC vs. bone marrow), donor matching type (MRD vs others) and HCT-CI (low vs high).
[b] Covariates for the analyses were: age, sex, conditioning regimen (MAC vs others), graft source (pBSC vs. bone marrow), donor matching type (MRD vs others), HCT-CI (low vs high) and for OS and NRM additionally aGvHD occurring before d+30 (No vs Yes).

A aGvHD incidence according to sex

Variable	Whole cohort (n = 442; 100%)	Females (n = 187; 42%)	Males (n = 255; 58%)	p-value
aGvHD incidence, n (%)				0.84
aGvHD grade 0 or 1	253 (57.5)	108 (58.1)	145 (57.1)	
aGvHD grade ≥2	187 (42.5)	78 (41.9)	109 (42.9)	

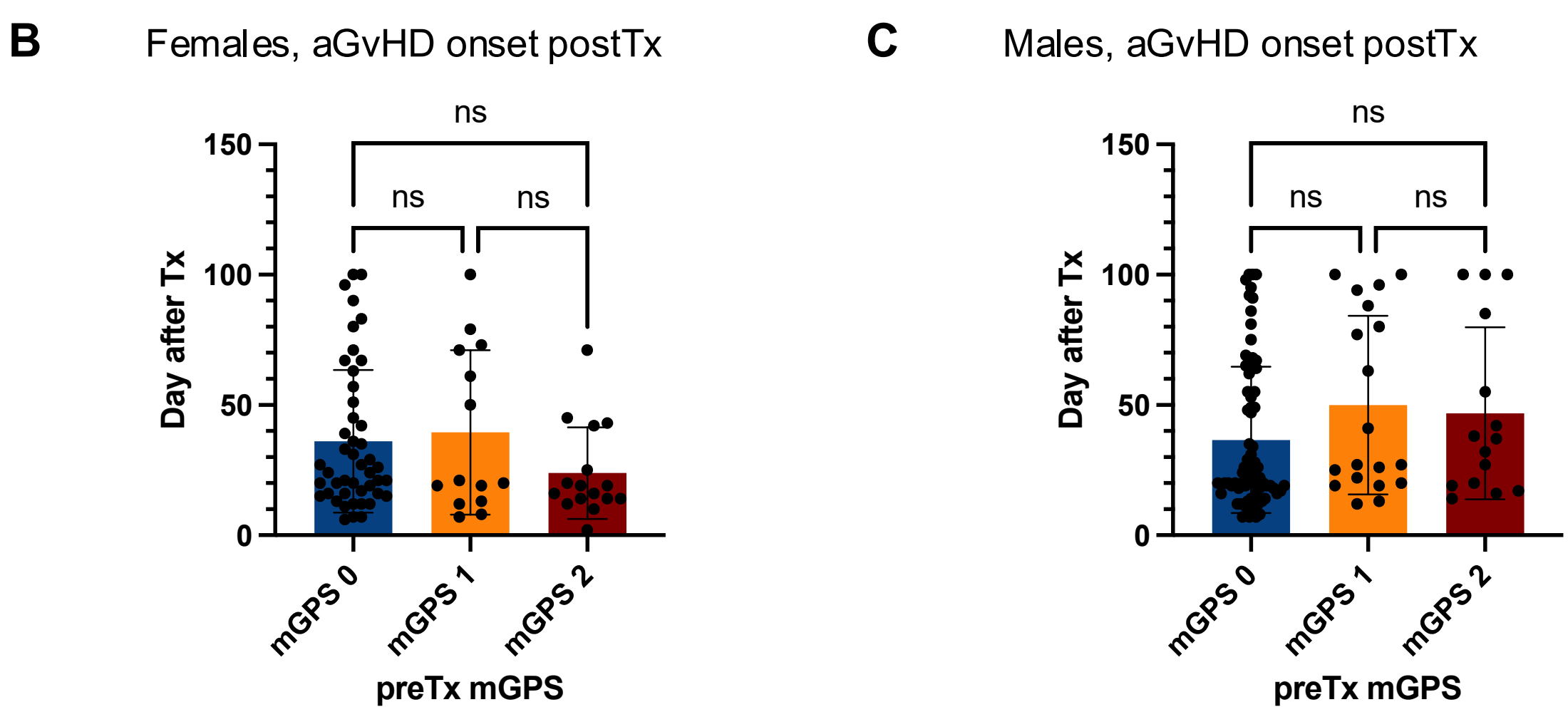

Supplementary Figure S2. Sex-specific analyses. (**A**) Incidence of aGvHD according to sex. (**B**) In all female and (**C**) in all male patients, scatter plots show the mean onset (days after Tx) of aGvHD grade ≥2 according to preTx mGPS.
Statistical differences were tested by the Kruskal-Wallis test.

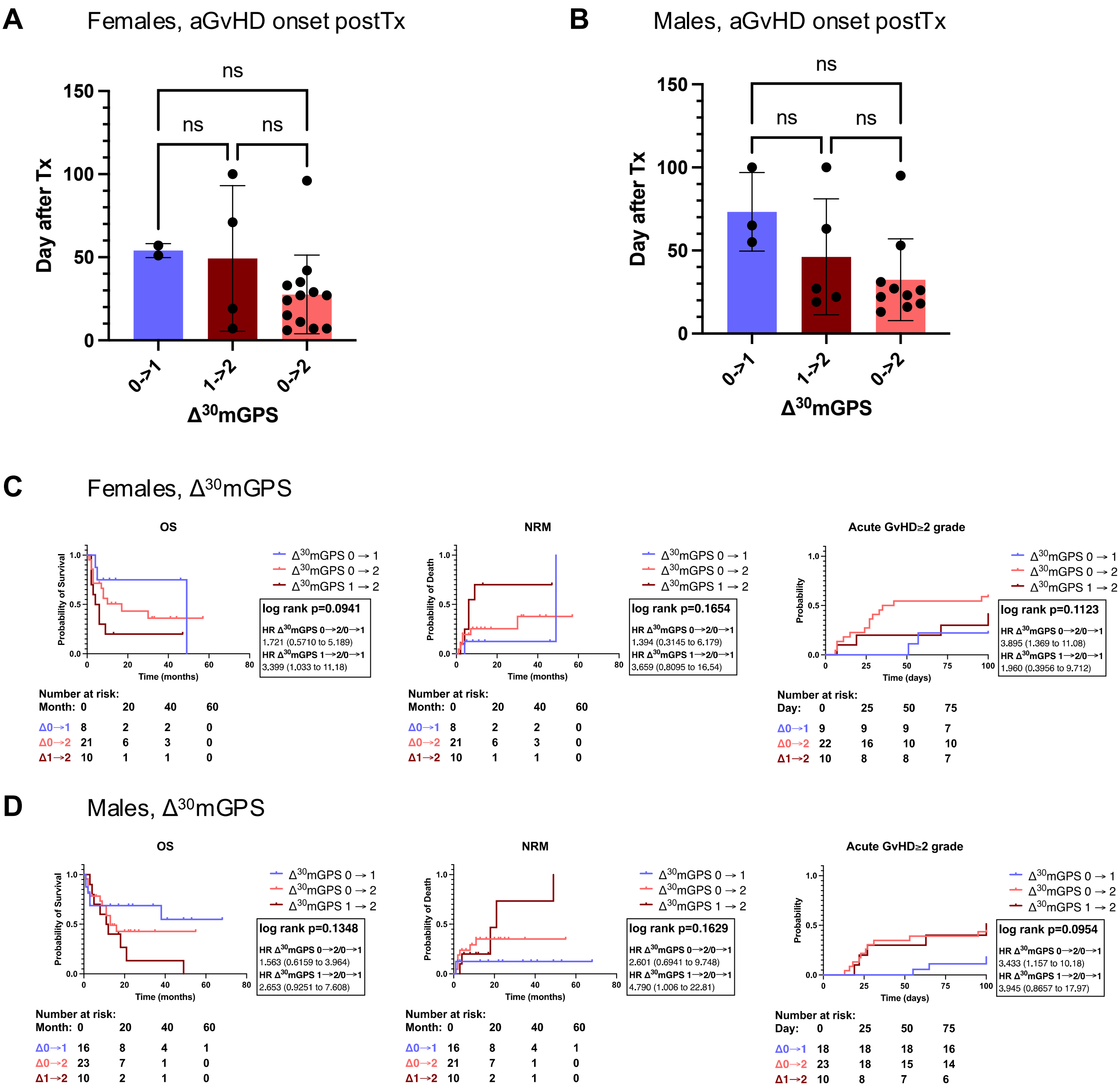

Supplementary Figure S3. Sex-specific analyses according to Δ^{30}mGPS.
(**A**) In all female and (**B**) in all male patients, scatter plots show the mean onset (days after Tx) of aGvHD grade ≥2 according to Δ^{30}mGPS. (**C**) For all females, Kaplan-Meier plots according to Δ30mGPS were generated addressing OS (n = 39), NRM (n = 39) and the cumulative incidence for aGvHD grade ≥2 (n = 41). (**D**) For all male patients, similar analyses according to Δ30mGPS were performed: OS (n = 49), NRM (n = 47) and aGvHD grade ≥2 (n = 51). Statistical analyses were performed with the log-rank test for survival curves and cumulative incidence of aGvHD. The Kruskal-Wallis test was employed to test differences in aGvHD onset.
